# The Influence of Modified Experimental Dental Resin Composites on the Initial In Situ Biofilm—A Triple-Blinded, Randomized, Controlled Split-Mouth Trial

**DOI:** 10.3390/polym13162814

**Published:** 2021-08-21

**Authors:** Niklas Burgard, Melanie Kienitz, Claudia Jourdan, Stefan Rüttermann

**Affiliations:** Department of Operative Dentistry, Faculty of Dental and Oral Medicine (Carolinum), Goethe-University Frankfurt am Main, 60323 Frankfurt, Germany; m.kienitz@med.uni-frankfurt.de (M.K.); jourdan@em.uni-frankfurt.de (C.J.); ruettermann@med.uni-frankfurt.de (S.R.)

**Keywords:** antibacterial composites, antiadhesive composites, Poly-Pore, split-mouth, clinical trial, live/dead staining, bacterial viability

## Abstract

The purpose of the study was to investigate the bacterial viability of the initial biofilm on the surface of experimental modified dental resin composites. Twenty-five healthy individuals with good oral hygiene were included in this study. In a split-mouth design, they received acrylic splints with five experimental composite resin specimens. Four of them were modified with either a novel polymeric hollow-bead delivery system or methacrylated polymerizable Irgasan (Antibacterial B), while one specimen served as an unmodified control (ST). A delivery system based on Poly-Pore^®^ was loaded with one of the active agents: Tego^®^ Protect 5000 (Antiadhesive A), Dimethicone (Antiadhesive B), or Irgasan (Antibacterial A). All study subjects refrained from toothbrushing during the study period. Specimens were detached from the splints after 8 h and given a live/dead staining before fluorescence microscopy. A Friedman test and a post hoc Nemenyi test were applied with a significance level at *p* < 0.05. In summary, all materials but Antibacterial B showed a significant antibacterial effect compared to ST. The results suggested the role of the materials’ chemistry in the dominance of cell adhesion. In conclusion, dental resin composites with Poly-Pore-loaded active agents showed antibacterial effectiveness in situ.

## 1. Introduction

A vast majority of dental fillings fail due to recurring carious lesions on the existing filling margins [1]. The development of this so-called secondary caries, in contrast to primary carious lesions without existing dental restorations, seems to depend on the filling properties to a large extend [2].

On one hand, it is comprehensible that the surface structure, in aspects of surface roughness or surface free energy of a dental filling, influences the bacterial adhesion, and consequently the development, of secondary caries [3,4,5,6,7,8,9]. On the other hand, it has been reported that the specific material itself can influence the caries formation. Accordingly, an amalgam is considered to be an effective filling material to modify the biofilm formation due to its bacteriostatic features [10]. In comparison, composite resin fillings show an increased plaque accumulation over the course of wearing [11], and fail more often than amalgams due to the development of secondary caries at the filling margins [12,13,14].

One strategy to prevent secondary caries could be to diminish or even inhibit bacterial adhesion [15,16,17], not only on the natural oral hard tissues, but also on the incorporated dental materials [18,19,20,21]. Therefore, innovative composite fillings with antiadhesive or antibacterial properties could play a key role in counteracting the risk of secondary caries.

For this purpose, our team developed and produced experimental resin composite materials that can release antibacterial or antiadhesive substances. The delivery process of these active substances as such is linked to comonomers or carrier substances, and is driven through abrasion processes [17,22,23,24]. Antiadhesive and antibacterial properties of these abrasion-responsive “smart materials” have already been observed in extensive in vitro studies [17,22,23,24,25].

To test the smart materials’ effect on the initial biofilm, some of these studies used the early colonizers as described by Kolenbrander et al. [26] in the form of monospecies cultures to show the influence on the number and viability of these bacterial strains with fluorescence microscopy examination [17,24]. As a result, the modified test materials were able to reduce the number of adherent bacteria in total and the proportion of vital to non-vital microorganisms [17,24].

The present study continued the aforementioned investigations in a randomized, triple-blinded, in situ split-mouth trial. This time, bacterial viability on the experimental but unmodified standard composite (ST) was compared with the most promising four experimental modified resin composites, Antiadhesive A (Poly-Pore-loaded Tego Protect 5000), Antiadhesive B (Poly-Pore-loaded dimethicone), Antibacterial A (Poly-Pore-loaded Irgasan) and Antibacterial B (polymerizable Methacryl-Irga). Hence, the aim of the present study was to clinically examine the effects known from in vitro studies in an in situ setting with subsequent fluorescence microscopy examination. The null hypothesis was that the modified materials would not differ from the control or among each other in the total bacterial counts or in the respective bacterium’s viability after 8 h.

## 2. Materials and Methods

### 2.1. Raw Materials and Its Modifications

Five experimental resin-based restorative materials were prepared (Table 1 and Table 2) using a laboratory vacuum mixer (Herbst Maschinenfabrik, Buxtehude, Germany).

The specifications of the composite materials and their manufacturing processes have been previously published, with the standard composite corresponding to material ST [17,22,24], and Antiadhesive A and Antiadhesive B corresponding to Material A and Material C, respectively [22,24]; and Antibacterial A and Antibacterial B corresponding to Material A and Material C, respectively [17].

The standard ST represented a common formulation for dental resin composites. The materials Antiadhesive A, Antiadhesive B, and Antibacterial A were produced by modifying ST by replacing glass filler parts with Poly-Pore sorption material. Antibacterial B was developed by modifying ST by replacing matrix parts with polymerizable Methacryl-Irga (Table 1).

The Poly-Pore delivery system for Antiadhesive A, Antiadhesive B, and Antibacterial A was manufactured by dissolving Tego Protect 5000, dimethicone, and Irgasan, respectively, in great excess of butanone (Lot 244238, Brenntag GmbH, Mühlheim, Germany) and adding Poly-Pore sorption material. The mixture was warmed slightly while stirring to evaporate the solvent. When the mixture became too stiff to stir, it was dried at 50 °C until a constant weight was reached. This procedure resulted in a completely dry and powdery Poly-Pore-based delivery system loaded with active agents.

### 2.2. Participants

The study was conducted in full accordance with the World Medical Association Declaration of Helsinki with the approval of the Ethics Committee of the Medical Faculty of Heinrich-Heine-University, Dusseldorf, Germany (internal study number: 2912). Written informed consent was obtained before each subject’s participation in the trial. The medical history was recorded and a dental report with tooth hard tissue status, periodontal condition, and oral hygiene was collected. The participants were evaluated for eligibility with the following inclusion criteria:

1. Age from 25 to 40 years;
2. Healthy dental condition;
3. No signs of periodontitis following the Periodontal Screening and Recording Index (PSR) [27];
4. Good oral hygiene within the limits of the Silness-Loe Plaque Index (PLI) [28];
5. No systemic diseases.

Subjects who did not meet the oral health parameters were offered to participate in a prophylaxis program and to have their carious lesions treated if any present. Participants were excluded if they did not meet the inclusion criteria.

### 2.3. Intervention

**Figure 1:**
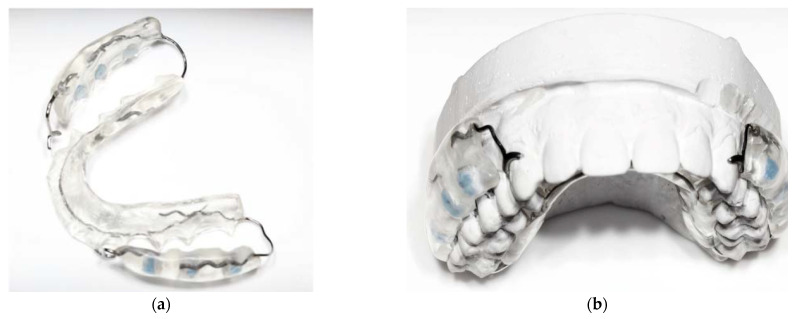
Custom-made removable acrylic splint: (**a**) specimens facing towards the buccal side of the first three approximal spaces of the posterior teeth; (**b**) placed onto a dental cast with the outward shielding element towards the cheeks and free space between the specimens and teeth, allowing salivatory flow.

Each participant received a removable custom-made acrylic splint that held the five specimens for simultaneous testing (Figure 1a). The specimens had to be inserted into depressions and fixed with sticky wax facing towards the buccal teeth surfaces at the level of the approximal spaces of the first three posterior teeth. This prevented the disruption of the biofilm caused by contact with the tongue or cheek on one hand, whereas the space between specimens and teeth remained free over a distance of 3 mm, allowing undisturbed biofilm growth and unhindered salivatory function on the other hand (Figure 1b).

### 2.4. Trial Design

The split-mouth design allowed the five specimens to be tested simultaneously in one run per subject. One specimen from the experimental unmodified composite material served as control, while the other four specimens were either antiadhesive- or antibacterial-modified experimental composite materials.

The specimens’ labels were encrypted by a third person, so that participant, clinical investigator, and laboratory evaluator were blinded throughout the study. In addition, the specimens’ assignment to the splint depressions by the clinical investigator and the later assessment of the specimens by the laboratory evaluator were randomized. The labels were only revealed again for statistical analysis.

Based on the preliminary in vitro study [24], sample size analysis was conducted for repeated measures ANOVA with a power of 80% using G*Power 3.1.9.2 [29]. Since means from ANOVA with multiple groups and equal group sizes were listed, the effect size Cohen’s f was translated from Cohen’s d using the difference between their smallest and largest mean over the pooled standard deviation [30]. Taking an intermediate variability of the mean dispersion over their range into account as proposed by Cohen [30], calculations were made based on the reported overall vital bacteria means and standard deviations after 8 h [24] for the relevant materials used in the present study. The significance level was set to α = 0.05, resulting in a total sample size of n = 25.

### 2.5. Specimen Preparation

Twenty-five disc-shaped specimens (diameter: 3 mm ± 0.1 mm; thickness: 1 mm ± 0.1 mm) from five experimental light-curing resin-based composites were made. The unmodified material ST, representing a common formulation of dental resin composites, served as the control. All materials met the ISO 4049 criteria [31]. The specimens were cured for 40 s on each side (Spectrum 800, Model No. 703EU, Dentsply DeTrey GmbH, Constance, Germany). The output of the curing device was checked routinely (Bluephase Meter, Ivoclar Vivadent AG, Schaan, Liechtenstein). Irradiances of 884 ± 53 mW/cm² were measured, and no significant decrease of the output was observed.

The cured specimens were polished on the test side with Super-Snap finishing and polishing discs (Schofu Dental GmbH, Ratingen, Germany), using green (20 µm grit) and red (7 µm grit) subsequently for one minute each at 10,000 rpm and a grinding pressure of 40–50 g.

### 2.6. Cell Viability Determination

After 8 h, the worn acrylic splints were removed, and the specimens were placed in 500 µL sterile 0.9% sodium chloride solution (Fresenius Kabi Deutschland GmbH, Bad Homburg, Germany). Afterwards, vital and non-vital cells were determined with live/dead staining (LIVE/DEAD^®^ BacLight Bacterial Viability Kit, Thermo Fisher Scientific GmbH, Dreieich, Germany) by measuring the fluorescence emission (BZ-X700E fluorescence microscope, Keyence Deutschland GmbH, Neu-Isenburg, Germany). The dye stock solution was prepared by mixing equal volumes of propidium iodide and SYTO9, and finally diluting 3 µL of the mixture with 1 mL 0.9% sodium chloride. Each specimen was finally incubated in 750 µL dye solution for 15 min.

Ten predetermined, randomly chosen locations were examined on each disc surface, and fluorescent microscopic images were captured (400-fold magnification) with fluorescent filter sets for both fluorescent dyes separately (SYTO9 480 nm, emission 500 nm; PI 490 nm, emission 635 nm). Specimens were processed randomly one after the other.

The absolute number of vital and non-vital cells and the sum of both were counted with the Hybrid Cell Count software (Keyence Deutschland GmbH) after haze reduction and black balance adjustment were applied. The bacterial cell viability ratio (BV) was reported as the percentage of vital cells from the total cell count.

### 2.7. Statistical Analysis

The medians and interquartile ranges were calculated and are presented as whisker-box plots with Tukey’s fences. Extreme values were considered for statistical analysis, but are not shown in the plots for reasons of clarity. The mean and standard deviation are also provided to compare the results with the results of previous studies. Normal distribution was tested using the Shapiro–Wilk test. As the data was not normally distributed, all statistical comparisons were performed using non-parametric methods. A Friedman test was applied to find differences between the composite groups. Post hoc pairwise comparisons were made using the conservative Nemenyi test, which already accounts for a familywise error [32]. Although no direct measure of effect size for the Friedman test is generally recognized, an indirect measure was obtained using the Kendall’s W-statistic, computed from the Friedman Q value [33]. Effect sizes were interpreted using Cohen’s interpretation guidelines [30]: small W < 0.3; moderate 0.3 ≤ W < 0.5; large W ≥ 0.5. Statistics and randomization processes were carried out with R software, version 4.0.5. The statistical significance level for all tests was set at *p* < 0.05.

## 3. Results

### 3.1. Participants

A total of 25 participants were selected from the catchment area of a German dental clinic for this split-mouth study. The participants’ characteristics are presented in Table 3.

In particular, the mean age of the included participants was 29.5 ± 3.3 years (median 29; range 25–39 years). They had no deceased teeth or signs of periodontitis (PSR ≤ 2). No participant showed a compromised oral hygiene (PLI ≤ 0.9).

### 3.2. Cell Viability

There were statistically significant differences in cell counts depending on the composite material tested. The effect sizes were moderate for the vital and total cell counts and the bacterial cell viability ratio BV (all *p* < 0.0001). The non-vital cell count showed a small effect (*p* = 0.00096).

**Figure 2:**
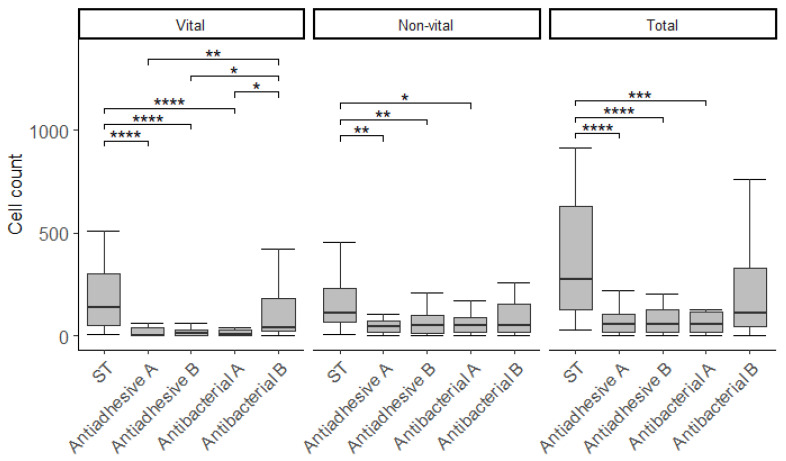
Tukey box plots of vital, non-vital, and total bacteria cell count without outliers. Significant differences are bracketed with asterisks (*, *p* < 0.05; **, *p* < 0.01; ***, *p* < 0.001; ****, *p* < 0.0001).

**Figure 3:**
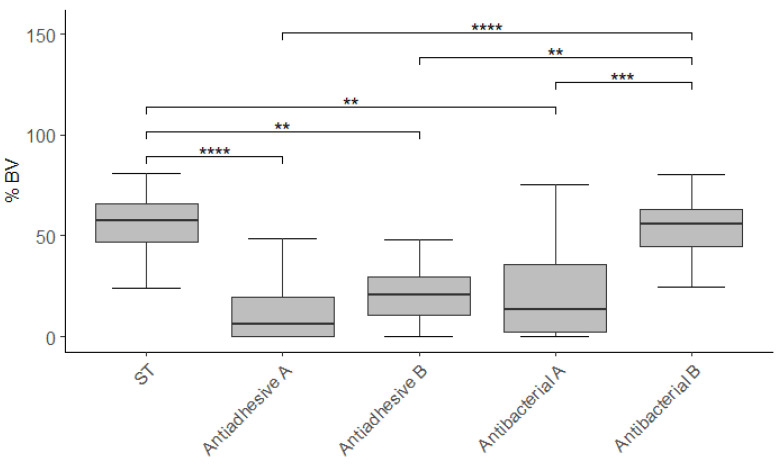
Tukey box plots of BV without outliers. Significant differences are bracketed with asterisks (*, *p* < 0.05; **, *p* < 0.01; ***, *p* < 0.001; ****, *p* < 0.0001).

The detailed results and the significances of the post hoc comparisons are shown in Table 4. The bacterial counts are additionally graphically presented in Figure 2 and Figure 3.

All materials but Antibacterial B showed significant fewer vital bacterial cells than ST (all *p* < 0.0001) (Figure 1). The Antibacterial B material had significantly more vital bacterial cells than the other modified materials (all *p* < 0.05).

Considering the non-vital bacterial cells, all test materials had significant fewer cells than ST (all *p* < 0.05) except Antibacterial B. The same could be observed for the total cell count, where all materials had fewer cells than ST except Antibacterial B (all *p* < 0.001).

A lower ratio of vital to total cells (BV) could be demonstrated for all materials but Antibacterial B in comparison to ST (all *p* < 0.01). The Antibacterial B material had a higher BV than the other modified test materials (all *p* < 0.01).

**Figure 4:**
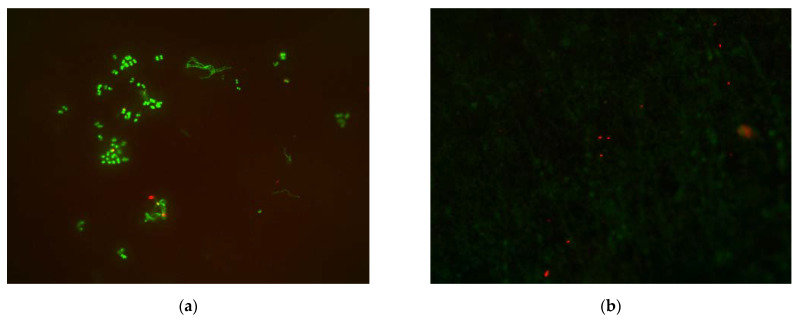
Comparison of representative superimposed fluorescence microscopic images (magnification 400-fold) of vital (green) and non-vital (red) bacterial cells from a single participant: (**a**) ST accumulated many vital and a few non-vital bacterial cells; (**b**) Antiadhesive A showed no vital but some non-vital bacterial microorganisms.

Representative fluorescence images are shown in Figure 4.

## 4. Discussion

The determination of cell viability by live/dead staining and subsequent measurement of the fluorescence emission is a common and established method [5,15,16,17,22,24,34,35,36,37].

ST and four modified experimental dental resin composites with appropriate flexural strength, flexural modulus, polymerization shrinkage, water sorption, solubility, contact angle θ, surface free energy (SFE), and biocompatibility of an author’s previous in-vitro studies were selected for their promising antibacterial effects [17,22,23,24,38]. All test materials were in accordance with the standard requested by EN ISO 4049 [31]. The preparation of ST and the modified materials by substituting the ST’s glass filler with a delivery system based on Poly-Pore [39] or by substituting the monomer matrix of ST with Methacryl-Irga [40,41,42] corresponded to the previously described procedure [17,22,24].

Consequently, ST and the modified materials Antiadhesive A, Antiadhesive B, or Antibacterial A did not differ in the type of matrix, but only in the substitution of filler parts by loaded Poly-Pore to release the active agents Tego Protect 5000, dimethicone, or Irgasan. The monomer matrix of the Antibacterial B material contained the polymerizable Methacryl-Irga as the only additive compared to ST. Due to the high irradiance of the light curing device [17,24,43,44,45] and a very low reported solubility (0.2 ± 0.8 to 1.0 ± 1.0 μg·mm^−3^) of all modified test materials and ST [17,22], an optimal polymerization could be expected [17,22,24,43,44,45,46,47]. Therefore, an antibacterial effect of the residual monomers was very unlikely, although the degree of polymerization was not measured [17,24]. In addition, there was no difference in polymerization shrinkage between the modified test materials and ST reported, which also indicated a good degree of conversion [17,22,48,49,50,51,52].

The specimens were polished in a standardized process to mimic the clinical situation and to activate the Poly-Pore-loaded active agents as described in previous studies [17,24]. The surface roughness $R_{a}$ of the polished materials’ specimens was analysed in previous studies, and no significant differences were found between the materials tested in the present study. Nevertheless, the influence of the surface roughness $R_{a}$ on bacterial adherence has been discussed thoroughly in the literature [4,5,6,7,8,20,53,54] and by an author [17,22,24]. In summary, $R_{a}$ ≤ 0.2 μm was judged to have a negligible effect [5,8,53,54]. In consequence, the $R_{a}$ of the polished materials’ specimens was assumed not to be a relevant factor in the present study based on the results of an author’s previous studies [17,22,24].

As we expected, our materials experienced the most interesting effect at the beginning of bacterial colonization, so the splint wearing time was limited to 8 h. The investigation of the test materials’ effect on cell viability at a very early stage of colonization was in accordance with an author’s previous in vitro studies [17,24].

The results presented in Table 4 demonstrate the antibacterial effects of both antiadhesive materials and Antibacterial A in comparison to ST. Although the previous in vitro studies with the investigation of antibacterial effects of the test materials on individual bacterial strains of the early colonizers A. naeslundii, A. viscosus, S. mitis, S. oralis, and S. sanguinis were sophisticated [17,24], they could only be partially observed clinically.

In the present study both antiadhesive materials and Antibacterial A showed significant fewer vital, non-vital, and total cells in comparison to ST. This effect could not be observed at all on the vital bacteria cells in previous in vitro studies for S. mitis, and hardly any for A. naeslundii [17,24]. Consequently, the role of S. mitis and partly of A. naeslundii in the early colonization of the mentioned materials can be questioned in the present study, given the fact that S. mitis with low total SFE $\gamma_{S}$ was reported to adhere better to low-$\gamma_{S}$ materials such as both antiadhesive test materials ($\gamma_{S}\;$≤ 29.9 ± 2.7 mJ·m^−2^) than to high-$\gamma_{S}$ materials like ST and Antibacterial B ($\gamma_{S}\;$≥ 42.9 ± 1.3 mJ·m^−2^) [24,55].

Regarding the in vitro results for non-vital and total cells, none of the three materials showed fewer cells than ST [17,24]. This demonstrated, to some degree, the antibacterial effects of the test materials’ modified surfaces on the bacterial cell adherence in the presence of saliva. The effect was very likely due to strong repulsive forces between the active agents and the aqueous oral medium, which quasi-forced the active agents to form a new thin, floating hydrophobic surface layer [22,24]. Under the given circumstances, the bacteria might not have been able to adhere directly to the materials’ surfaces, but only to the floating layer, and therefore they might have been washed off by saliva, which was not detected in vitro. The lack of improvement of Antibacterial B with polymerized Methacryl-Irga in the present study compared to the other modified test materials with Poly-Pore-loaded agents supported this hypothesis. On one hand, Antibacterial B showed a clear antibacterial effect in vitro on the cell viability for most of the early colonizers compared to ST [24], which could not be observed in the present study. On the other hand, there were no differences between the in vitro results regarding non-vital and total cells for most of the colonizers [24] and the results of the present study.

In addition, the lack of correlation between the reported contact angle θ [17,22] and the test materials’ total bacterial counts in the present study supported the assumption that the material chemistry dominated cell adhesion [24]. The association of θ and bacterial adhesion has already been extensively discussed in previous studies [17,24,56,57,58]. Overall composite resins are assumed to be more resistant against attack by water or water-soluble species with higher hydrophobicity [58,59,60]. Contrarily, it was also hypothesized that hydrophobic surfaces would support the cell adhesion by removing water more easily between bacterial cells and the material, and thus allowing a closer approach with stronger adhesive forces between the cell surface and hydrophilic material [56]. However, compared to ST, both antibacterial materials did not show statistically significant different contact angles θ [17]. Nevertheless, Antibacterial A had significantly lower bacterial counts in the present study, allowing a conclusion regarding the materials’ chemical influence. It should also be noted that the two antiadhesive test materials were the only ones with previously measured significant lower total SFE $\gamma_{S}$ than ST (both $\gamma_{S}$ < 30 ${\mathrm{mJ}\;m}^{-2}$) [22], and thus according to Vogler’s interpretation, hydrophobic by definition [61], which currently resulted in fewer cells for these materials. This coincided with in vivo studies that showed low supragingival plaque formation and thus low adhesion and biofilm formation for low $\gamma_{S}$ substrata [5,9].

Furthermore, taking the reported polar $\gamma_{S}^{AB}$ values of the SFE into account, all materials ($\gamma_{S}^{AB}$ between −2.4 ± 1.3 mJ·m^−2^ and −0.8 ± 0.7 mJ·m^−2^) but Antibacterial B ($\gamma_{S}^{AB}$ 4.3 ± 1.7 mJ·m^−2^) were reported to have significantly lower values than ST ($\gamma_{S}^{AB}$ 3.7 ± 2.0 mJ·m^−2^) [17,22]. High polar term $\gamma_{S}^{AB}$ was found to create strong bacterial adhesion, which implied that the low $\gamma_{S}^{AB}$ might have reduced bacterial adhesion for all the modified test materials but Antibacterial B [19,24,62].

All in all, biofilm formation is very complex and does not only include bacterial interaction. Therefore, protein adhesion on pellicle-coated surfaces should also be investigated in further studies. In addition, the comparison of previous in vitro results with the present results was limited because numerous interactions may have occurred in the oral cavity that may have influenced the results, and were not followed up.

## 5. Conclusions

The present study demonstrated the protective effect of experimental dental resin composites modified with small amounts of a novel antiadhesive or antibacterial loaded into a delivery system. The sorption material, being part of the delivery system, might be used as a vehicle for any other, and perhaps an even more effective, active agent. Based on the results of the study, the null hypothesis must be rejected for all test materials but Antibacterial B, as they showed significant differences with the unmodified control composite resin ST.

## Figures and Tables

**Table 1:** Formulations of the experimental resin-based restorative materials and fraction of effective active agent in total mass. ST served as control (all data in wt %).

**Raw Material**	ST	Antiadhesive A	Antiadhesive B	Antibacterial A	Antibacterial B
Glass	73.0	68.0	68.2	68.0	73.0
Poly-Tego	-	5.0	-	-	-
Poly-Dimeth	-	-	5.0	-	-
Poly-Irga	-	-	-	5.0	-
Methacryl-Irga	-	-	-	-	8.0
Matrix	27.0	27.0	26.8	27.0	19.0
**Total**	100	100	100	100	100
Active agent	0	4.0	4.0	4.0	8.0

Matrix: UDMA, 44.1; Bis-GMA, 30.0; TTEGDMA, 25.0; photonitiator, 0.3; CQ, 0.2; amine, 0.1; stabilizer, 0.1.

**Table 2:** The raw materials, based on the manufacturers’ technical data sheets.

Code	Product/Properties	Batch	Company
Photoinitiator	α.α-dimethoxy-α-phenylacetophenone	0066162S	Ciba Specialty Chemicals, Basel, Switzerland
Stabilizer	Pentaerythrityl-tetrakis[3-(3,5-di-tert-butyl-4-hydroxyphenyl)-propionate	26099IC3	Ciba Speciality Chemicals
TTEGDMA	Tetraethyleneglycole dimethacrylate, standard monomer, functionality = 2, MW = 330 g·mol^−1^, good chemical and physical properties, very low viscosity (14 Pa s, 25 °C), diluting	J1620	Cray Valley, Paris, France
UV stabilizer	2-Hydroxy-4-methoxy-bezophenone	411351/ 143302	Fluka, Buchs, Switzerland
UDMA	7,7,9-Trimethyl-4,13-dioxo-3,14-dioxa-5,12-diaza-hexadecan-1,16-diol-dimethacrylate, standard monomer, functionality = 2, MW = 471 g·mol^−1^, flexible, tough, very good chemical resistance, medium viscosity(10,000 m·Pas,25 °C)	330503057	Rahn A.G, Zürich, Switzerland
Bis-GMA	Bis-GMA, standard monomer, functionality = 2, MW = 513 g·mol^−1^, rigid, very good chemical resistance, very high viscosity (4500 m·Pas, 60 °C)	2008218303	Rahn A.G
CQ	D,L-Camphorquinone	0148990002	Rahn A.G
Amine	Ethyl-4-(dimethylamino)-benzoate	310170	Rahn A.G
Glass	Strontium borosilicate glass (GO 18–093, d50 = 0.7 µm). silaned (3-methacryloyloxypropyl trimethoxy silane), D = 2.6 g·cm^−3^,	Lab14701	Schott Electronic Packaging, GmbH, Landshut, Germany
Poly-	Poly-Pore, cross-linked polyallyl methacrylate, adsorber, hollow beads, diameter 20–40 µm	L07070303AB	AMCOL Health & Beauty Solutions, Arlington Heights, IL, USA
Tego	Tego Protect 5000, hydroxyfunctional polydimethylsiloxane, hydro- and oleophobic, D = 1.05 g·cm^−3^	ES57608918	Evonik Tego Chemie, Essen, Germany
Dimeth	Dimethicone 200/350 cst, polydimethylsiloxane, D = 0.965 g·cm^−3^	4962250	Dow Corning Corp., Midland, MI, USA
Irga	Irgasan, 5-chloro-2-(2,4-dichlorophenoxy)phenole	1124816	Sigma Aldrich GmbH, Steinheim, Germany
Poly-Dimeth	Poly-Pore loaded with 80% dimethicone, D = 1.0 g·cm^−3^	Experimental product	University laboratory
Poly-Tego	Poly-Pore loaded with 80% Tego Protect 5000, D = 1.0 g·cm^−3^	Experimental product	University laboratory
Poly-Irga	loaded with 80% Irgasan, D = 1.0 cm^−3^	Experimental product	University laboratory
Methacryl-Irga	5-chloro-2-(2,4-dichlorophenoxy)phenyl methacrylate	Experimental product	University laboratory

**Table 3:** The study participants’ characteristics.

	n	%
Participants	25	100
Female/Male	19/6	76/24
Age (mean ± SD) (years)	29.5 ± 3.3	-
Tooth hard tissue		
Decay	0	-
Oral hygiene (PLI)		
Excellent (0)	16	64.0
Good (0.1–0.9)	9	36.0
Fair (1.0–1.9)	0	-
Poor (2.0–3.0)	0	-
Periodontal Screening and Recording Index (PSR)		
Grade 0	104	69.3
Grade 1	27	18
Grade 2	19	12.7
Grade 3	0	-
Grade 4	0	-

Abbreviations: n, number; SD, standard deviation.

**Table 4:** Cell count medians (and interquartile ranges) of vital, non-vital, and total cells, and the bacterial cell viability ratio (BV). Means ± standard deviations are provided in square brackets. Values are rounded to valid digits. Equal subscript numbers within the columns indicate *non-*significant differences between the materials (*p* > 0.05).

Material	n	Vital	Non-Vital	Total	% BV
ST	25	137.7 (251.4)_1_	108.5 (163.4)_1_	276.2 (506.4)_1_	57.6 (19.4)_1_
		[463.7 ± 913.2]	[274.4 ± 540.0]	[738.1 ± 1434.2]	[55.2 ± 18.7]
Antiadhesive A	25	2.7 (37.0)_2_	44.0 (53.9)_2_	57.7 (84.9)_2_	6.4 (19.7)_2_
		[66.6 ± 235.3]	[135.4 ± 286.1]	[202.0 ± 507.6]	[15.3 ± 20.8]
Antiadhesive B	25	10.8 (27.0)_2_	48.1 (89.2)_2_	54.1 (113.0)_2_	20.6 (18.9)_2_
		[141.9 ± 604.2]	[132.8 ± 345.6]	[274.7 ± 946.9]	[23.6 ± 22.2]
Antibacterial A	25	5.0 (26.9)_2_	50.9 (70.6)_2_	53.3 (96.3)_2_	13.2 (33.6)_2_
		[105.0 ± 321.7]	[182.0 ± 429.0]	[287.0 ± 712.2]	[22.2 ± 23.0]
Antibacterial B	25	41.6 (160.4)_1_	52.5 (138.3)_1,2_	111.6 (286.9)_1,2_	55.6 (18.4)_1_
		[298.7 ± 926.5]	[206.6 ± 620.0]	[505.3 ± 1545.3]	[51.9 ± 17.7]

Abbreviations: n, number; ST, unmodified material (control).

## Data Availability

The data presented in this study are available on request from the corresponding author. The data are not publicly available due to institutional data protection regulations.

## References

[1] Mjor I.A., Toffenetti F. (2000). Secondary caries: A literature review with case reports. Quintessence Int..

[2] Ruben J., Arends J., Christoffersen J. (1999). The effect of window width on the demineralization of human dentine and enamel. Caries Res..

[3] Hao Y., Huang X., Zhou X., Li M., Ren B., Peng X., Cheng L. (2018). Influence of Dental Prosthesis and Restorative Materials Interface on Oral Biofilms. Int. J. Mol. Sci.

[4] Quirynen M., Bollen C.M. (1995). The influence of surface roughness and surface-free energy on supra- and subgingival plaque formation in man. A review of the literature. J. Clin. Periodontol..

[5] Quirynen M., Marechal M., Busscher H.J., Weerkamp A.H., Darius P.L., van Steenberghe D. (1990). The influence of surface free energy and surface roughness on early plaque formation. An in vivo study in man. J. Clin. Periodontol..

[6] Aykent F., Yondem I., Ozyesil A.G., Gunal S.K., Avunduk M.C., Ozkan S. (2010). Effect of different finishing techniques for restorative materials on surface roughness and bacterial adhesion. J. Prosthet. Dent..

[7] Beyth N., Bahir R., Matalon S., Domb A.J., Weiss E.I. (2008). Streptococcus mutans biofilm changes surface-topography of resin composites. Dent. Mater..

[8] Bollen C.M., Lambrechts P., Quirynen M. (1997). Comparison of surface roughness of oral hard materials to the threshold surface roughness for bacterial plaque retention: A review of the literature. Dent. Mater..

[9] Quirynen M., Marechal M., Busscher H.J., Weerkamp A.H., Arends J., Darius P.L., van Steenberghe D. (1989). The influence of surface free-energy on planimetric plaque growth in man. J. Dent. Res..

[10] Morrier J.J., Suchett-Kaye G., Nguyen D., Rocca J.P., Blanc-Benon J., Barsotti O. (1998). Antimicrobial activity of amalgams, alloys and their elements and phases. Dent. Mater..

[11] Shahal Y., Steinberg D., Hirschfeld Z., Bronshteyn M., Kopolovic K. (1998). In vitro bacterial adherence onto pellicle-coated aesthetic restorative materials. J. Oral Rehabil..

[12] van de Sande F.H., Opdam N.J.M., Truin G.J., Bronkhorst E.M., de Soet J.J., Cenci M.S., Huysmans M.-C. (2014). The influence of different restorative materials on secondary caries development in situ. J. Dent..

[13] Opdam N.J.M., Bronkhorst E.M., Loomans B.A.C., Huysmans M.-C.D.N.J.M. (2010). 12-year Survival of Composite vs. Amalgam Restorations. J. Dent. Res..

[14] Bernardo M., Luis H., Martin M.D., Leroux B.G., Rue T., Leitão J., DeRouen T.A. (2007). Survival and reasons for failure of amalgam versus composite posterior restorations placed in a randomized clinical trial. J. Am. Dent. Assoc..

[15] Al-Ahmad A., Follo M., Selzer A.C., Hellwig E., Hannig M., Hannig C. (2009). Bacterial colonization of enamel in situ investigated using fluorescence in situ hybridization. J. Med. Microbiol..

[16] Jung D.J., Al-Ahmad A., Follo M., Spitzmuller B., Hoth-Hannig W., Hannig M., Hannig C. (2010). Visualization of initial bacterial colonization on dentine and enamel in situ. J. Microbiol. Methods.

[17] Rüttermann S., Trellenkamp T., Bergmann N., Beikler T., Ritter H., Janda R. (2013). Bacterial viability and physical properties of antibacterially modified experimental dental resin composites. PLoS ONE.

[18] Burgers R., Hahnel S., Reichert T.E., Rosentritt M., Behr M., Gerlach T., Handel G., Gosau M. (2010). Adhesion of Candida albicans to various dental implant surfaces and the influence of salivary pellicle proteins. Acta Biomater..

[19] Knorr S.D., Combe E.C., Wolff L.F., Hodges J.S. (2005). The surface free energy of dental gold-based materials. Dent. Mater..

[20] Teughels W., Van Assche N., Sliepen I., Quirynen M. (2006). Effect of material characteristics and/or surface topography on biofilm development. Clin. Oral Implant. Res..

[21] Wiegand A., Buchalla W., Attin T. (2007). Review on fluoride-releasing restorative materials--fluoride release and uptake characteristics, antibacterial activity and influence on caries formation. Dent. Mater..

[22] Rüttermann S., Trellenkamp T., Bergmann N., Raab W.H., Ritter H., Janda R. (2011). A new approach to influence contact angle and surface free energy of resin-based dental restorative materials. Acta Biomater..

[23] Rüttermann S., Beikler T., Janda R. (2014). Contact angle and surface free energy of experimental resin-based dental restorative materials after chewing simulation. Dent. Mater..

[24] Rüttermann S., Bergmann N., Beikler T., Raab W.H., Janda R. (2012). Bacterial viability on surface-modified resin-based dental restorative materials. Arch. Oral Biol..

[25] Henrich B., Hermann I., Di Giulio M., Köhrer K., Deenen R., Sivalingam S., Peters U., Beikler T., Janda R., Rüttermann S. (2016). Reexamination In Vitro and In Situ of an Antibacterially Modified Experimental Dental Resin Composite with Molecular Methods: A Pilot Study. Adv. Mater. Sci. Eng..

[26] Kolenbrander P.E., Palmer R.J., Periasamy S., Jakubovics N.S. (2010). Oral multispecies biofilm development and the key role of cell-cell distance. Nat. Rev. Microbiol..

[27] American Dental Association (1993). Periodontal screening & recording an early detection system. J. N. J. Dent. Assoc..

[28] Silness J., Loe H. (1964). Periodontal disease in pregnancy. Ii. Correlation between oral hygiene and periodontal condtion. Acta Odontol. Scand..

[29] Faul F., Erdfelder E., Lang A.-G., Buchner A. (2007). G*Power 3: A flexible statistical power analysis program for the social, behavioral, and biomedical sciences. Behav. Res. Methods.

[30] Cohen J. (1988). Statistical Power Analysis for the Behavioral Sciences.

[31] International Organization for Standardization (2009). ISO 4049: Dentistry-Polymer-Based Filling, Restortive and Luting Materials.

[32] Sachs L. (1997). Angewandte Statistik.

[33] Tomczak M., Tomczak E. (2014). The need to report effect size estimates revisited. An overview of some recommended measures of effect size. Trends Sport Sci..

[34] Al-Ahmad A., Wunder A., Auschill T.M., Follo M., Braun G., Hellwig E., Arweiler N.B. (2007). The in vivo dynamics of *Streptococcus* spp., *Actinomyces naeslundii*, *Fusobacterium nucleatum* and *Veillonella* spp. in dental plaque biofilm as analysed by five-colour multiplex fluorescence in situ hybridization. J. Med. Microbiol..

[35] Hannig C., Follo M., Hellwig E., Al-Ahmad A. (2010). Visualization of adherent micro-organisms using different techniques. J. Med. Microbiol..

[36] Hannig C., Hannig M., Rehmer O., Braun G., Hellwig E., Al-Ahmad A. (2007). Fluorescence microscopic visualization and quantification of initial bacterial colonization on enamel in situ. Arch. Oral Biol..

[37] ten Cate J.M. (2006). Biofilms, a new approach to the microbiology of dental plaque. Odontology.

[38] Landenberger P., Baumann L., Gerhardt-Szép S., Rüttermann S. (2021). The effect of new anti-adhesive and antibacterial dental resin filling materials on gingival fibroblasts. Dent. Mater..

[39] Sojka M.F. (1998). Precipitation Polymerization Process for Producing an Oil Adsorbent Polymer Capable of Entrapping Solid Particles and Liquids and the Product Thereof. U.S. Patent.

[40] Oh S.T., Han S.H., Ha C.S., Cho W.J. (1996). Synthesis and biocidal activities of polymer. IV. Antibacterial activity and hydrolysis of polymers containing diphenyl ether. J. Appl. Polym. Sci..

[41] Oh S.T., Ha C.S., Cho W.J. (1994). Synthesis and biocidal activities of polymer. III. Bactericical activity of homopolymer of AcDP and copolymer of acdp with St. J. Appl. Polym. Sci..

[42] Choi S.-b., Jepperson J., Jarabek L., Thomas J., Chisholm B., Boudjouk P. (2007). Novel Approach to Anti-Fouling and Fouling-Release Marine Coatings Based on Dual-Functional Siloxanes. Macromol. Symp..

[43] Rüttermann S., Krüger S., Raab W.H., Janda R. (2007). Polymerization shrinkage and hygroscopic expansion of contemporary posterior resin-based filling materials—a comparative study. J. Dent..

[44] Janda R., Roulet J.F., Latta M., Rüttermann S. (2007). Water sorption and solubility of contemporary resin-based filling materials. J. Biomed. Mater. Res. Part B Appl. Biomater..

[45] Janda R., Roulet J.F., Latta M., Rüttermann S. (2006). The effects of thermocycling on the flexural strength and flexural modulus of modern resin-based filling materials. Dent. Mater..

[46] da Silva E.M., Almeida G.S., Poskus L.T., Guimarães J.G. (2008). Relationship between the degree of conversion, solubility and salivary sorption of a hybrid and a nanofilled resin composite. J. Appl. Oral Sci..

[47] Gonçalves L., Filho J.D., Guimarães J.G., Poskus L.T., Silva E.M. (2008). Solubility, salivary sorption and degree of conversion of dimethacrylate-based polymeric matrixes. J. Biomed. Mater. Res. Part B Appl. Biomater..

[48] Cho E., Sadr A., Inai N., Tagami J. (2011). Evaluation of resin composite polymerization by three dimensional micro-CT imaging and nanoindentation. Dent. Mater..

[49] Feng L., Suh B.I. (2006). The effect of curing modes on polymerization contraction stress of a dual cured composite. J. Biomed. Mater. Res. Part B Appl. Biomater..

[50] Sharifi S., Mirzadeh H., Imani M., Atai M., Ziaee F. (2008). Photopolymerization and shrinkage kinetics of in situ crosslinkable N-vinyl-pyrrolidone/poly(ε-caprolactone fumarate) networks. J. Biomed. Mater. Res. Part A.

[51] Sideridou I.D., Karabela M.M., Micheliou C.N., Karagiannidis P.G., Logothetidis S. (2009). Physical properties of a hybrid and a nanohybrid dental light-cured resin composite. J. Biomater. Sci. Polym. Ed..

[52] Silikas N., Eliades G., Watts D.C. (2000). Light intensity effects on resin-composite degree of conversion and shrinkage strain. Dent. Mater..

[53] Quirynen M., Bollen C.M., Papaioannou W., Van Eldere J., van Steenberghe D. (1996). The influence of titanium abutment surface roughness on plaque accumulation and gingivitis: Short-term observations. Int. J. Oral Maxillofac. Implant..

[54] Bollen C.M., Papaioanno W., Van Eldere J., Schepers E., Quirynen M., van Steenberghe D. (1996). The influence of abutment surface roughness on plaque accumulation and peri-implant mucositis. Clin. Oral Implant. Res..

[55] Uyen M., Busscher H.J., Weerkamp A.H., Arends J. (1985). Surface free energies of oral streptococci and their adhesion to solids. FEMS Microbiol. Lett..

[56] Mei L., Busscher H.J., van der Mei H.C., Chen Y., de Vries J., Ren Y. (2009). Oral bacterial adhesion forces to biomaterial surfaces constituting the bracket-adhesive-enamel junction in orthodontic treatment. Eur. J. Oral Sci..

[57] Gyo M., Nikaido T., Okada K., Yamauchi J., Tagami J., Matin K. (2008). Surface response of fluorine polymer-incorporated resin composites to cariogenic biofilm adherence. Appl. Environ. Microbiol..

[58] Buergers R., Schneider-Brachert W., Hahnel S., Rosentritt M., Handel G. (2009). Streptococcal adhesion to novel low-shrink silorane-based restorative. Dent. Mater..

[59] Eick J.D., Kotha S.P., Chappelow C.C., Kilway K.V., Giese G.J., Glaros A.G., Pinzino C.S. (2007). Properties of silorane-based dental resins and composites containing a stress-reducing monomer. Dent. Mater..

[60] Weinmann W., Thalacker C., Guggenberger R. (2005). Siloranes in dental composites. Dent. Mater..

[61] Vogel B.S., Wildung M.R., Vogel G., Croteau R. (1996). Abietadiene synthase from grand fir (*Abies grandis*). cDNA isolation, characterization, and bacterial expression of a bifunctional diterpene cyclase involved in resin acid biosynthesis. J. Biol. Chem..

[62] Lee S.P., Lee S.J., Lim B.S., Ahn S.J. (2009). Surface characteristics of orthodontic materials and their effects on adhesion of mutans streptococci. Angle Orthod..

